# Efficacy of tPBM on ADHD symptoms and Executive Function Deficits in Adults with high-functioning Autism Spectrum Disorder

**DOI:** 10.1192/j.eurpsy.2022.2248

**Published:** 2022-09-01

**Authors:** T.A. Ceranoglu, P. Cassano, C. Hutt Vater, A. Green, N. Dallenbach, M. Disalvo, J. Biederman, G. Joshi

**Affiliations:** Massachusetts General Hospital, Psychiatry, Boston, United States of America

**Keywords:** Transcranial Photobiomodulation, Autism Spectrum Disorder, attention-deficit/hyperactivity disorder

## Abstract

**Introduction:**

Executive function (EF) deficits are often associated with Autism Spectrum Disorder (ASD), even in the absence of Attention Deficit Hyperactivity Disorder (ADHD) diagnosis. To date, no approved medication treatments exist for EF deficits associated with ASD.

**Objectives:**

To assess the efficacy of transcranial photobiomodulation (tPBM) on EF in adults with ASD.

**Methods:**

Adults (18-59) with high-functioning (HF)-ASD received twice a week tPBM for 8 weeks in an open-label single group design. ASD and EF deficits were assessed by clinician-rated Clinical Global Impression Scale and patient-rated scales of Behavior Rating Inventory of Executive Function-Adult (BRIEF-A).

**Results:**

Eleven participants were enrolled. Ten participants completed the study. Nine participants who completed the study had comorbid ADHD diagnosis. All 10 participants were included in efficacy analyses of EF deficits. Statistically significant improvements in executive function deficits were found in BRIEF-A total score and in subdomains of Inhibition, Emotional Control, Planning and Organization, Organization of Materials, Behavioral Regulation, Metacognitive Index and Global Executive Control. All participants were found to have mild to moderate improvement in their ADHD symptom severity per clinician rated CGIs. Statistically significant improvements in ADHD symptoms were noted in self-rated scales. No adverse events required changes in tPBM protocol.

**Conclusions:**

tPBM is a safe and feasible treatment approach that has the potential to treat core features of ASD. Further research is necessary and warranted.

**Disclosure:**

This work is funded by Alan and Lorraine Bressler Clinical and Research Program for Autism Spectrum Disorder and the MGH Pediatric Psychopharmacology Council Fund.

